# A feasibility and safety study of afamelanotide in acute stroke patients – an open label, proof of concept, phase iia clinical trial

**DOI:** 10.1186/s12883-023-03338-9

**Published:** 2023-07-26

**Authors:** Vimal Stanislaus, Anthony Kam, Lily Murphy, Philippe Wolgen, Gill Walker, Pilar Bilbao, Geoffrey C Cloud

**Affiliations:** 1 Department of Neuroscience, Central Clinical School, Monash University, Melbourne, Australia; 2 Alfred Hospital, Melbourne, Australia; 3 CLINUVEL Pharmaceuticals, Melbourne, Australia

**Keywords:** Acute ischemic stroke, Neuroprotective, Afamelanotide, Melanocyte stimulating hormone

## Abstract

**Background:**

Neuroprotective agents have the potential to improve the outcomes of revascularisation therapies in acute ischemic stroke patients (AIS) and in those unable to receive revascularisation. Afamelanotide, a synthetic α-melanocyte stimulating hormone analogue, is a potential novel neuroprotective agent. We set out to assess the feasibility and safety of afamelanotide for the first time in AIS patients.

**Methods:**

AIS patients within 24 h of onset, with perfusion abnormality on imaging (Tmax) and otherwise ineligible for revascularisation therapies were enrolled. Afamelanotide 16 mg implants were administered subcutaneously on Day 0 (D0, day of recruitment), D1 and repeated on D7 and D8, if not well recovered. Treatment emergent adverse events (TEAEs) and neurological assessments were recorded regularly up to D42. Magnetic resonance imaging (MRI) with FLAIR sequences were also performed on D3 and D9.

**Results:**

Six patients (5 women, median age 81, median NIHSS 6) were recruited. Two patients received 4 doses and four patients received 2. One patient (who received 2 doses), suffered a fatal recurrent stroke on D9 due to a known complete acute internal carotid artery occlusion, assessed as unrelated to the study drug. There were no other local or major systemic TEAEs recorded. In all surviving patients, the median NIHSS improved from 6 to 2 on D7. The median Tmax volume on D0 was 23 mL which was reduced to a FLAIR volume of 10 mL on D3 and 4 mL on D9.

**Conclusions:**

Afamelanotide was well tolerated and safe in our small sample of AIS patients. It also appears to be associated with good recovery and radiological improvement of salvageable tissue which needs to be tested in randomized studies.

**ClinicalTrials.gov Identifier:**

NCT04962503, First posted 15/07/2021.

## Introduction

Stroke is a leading cause of mortality and morbidity worldwide [1]. Acute ischemic stroke (AIS) accounts for 85% of all strokes and is commonly associated with thrombotic obstruction in cerebral blood vessels causing reduced blood flow to the affected part of the brain and cerebral ischemia. Ischemia disrupts the blood brain barrier (BBB) by activating proteinases such as matrix metalloproteinases (MMPs) and altering proteins in the BBB tight junction such as integrins. This triggers a cascade of inflammatory reactions causing further damage to the ischemic tissue.

Current revascularisation therapies in AIS include thrombolysis and endovascular clot retrieval. They are aimed at removing the thrombotic obstruction and retaining blood flow to the ischemic tissue but cannot prevent injury to the BBB or inflammatory reactions. They are also restricted by several limitations. Both are time dependent, limited by patient selection, need specially trained professional and available only in selected centres. Only about 10–20% of AIS patients would qualify for these therapies and of those only 30–40% of patients will achieve functional independence [2].

Neuroprotective agents reduce inflammation, may repair or minimise the damage to the BBB and thus can protect neurons. They also have the potential not only to improve the eligibility but also the outcomes of stroke revascularisation therapies and in those unable to receive revascularisation. However, despite more than 1000 pre-clinical studies and over 200 clinical trials, no effective neuroprotective agent has been found [3–6]. Animals used in most of these studies lack the heterogeneity seen in stroke patients. In addition to that, only few of these agents were tested in acute stroke.

The neuropeptide hormones α-melanocyte stimulating hormone (α-MSH) and melanocortin are favourably implicated in AIS for their neuroprotective effects and strong anti-inflammatory properties [7–10]. Animal studies have shown that α-MSH levels rapidly decrease following arterial occlusion in AIS [11]. Patients with acute brain injuries including AIS and traumatic brain injury have also been found to have decreased α-MSH levels [12]. Lower α-MSH levels post AIS were associated with severe stroke and worse outcomes while higher α-MSH levels were associated with good long term outcomes [10, 12]. Multiple animal studies have shown that exogenous α-MSH provides long lasting protection against ischemia, decreases infarct volume and improves stroke outcomes [11, 13–17].

Afamelanotide is a synthetic, highly potent, non-selective agonist of α-MSH analogue, currently licenced for erythropoietic protoporphyria (EPP). Ample evidence is available from clinical studies that it is generally safe and well tolerated [18, 19]. Existing animal studies on administration of α-MSH analogue post AIS suggests that the anti-inflammatory and neuroprotective responses of melanocortins are dose dependent. Improvement in functional recovery and decrease in infarct volume were seen when α-MSH was given early post stroke and at higher doses [11, 14, 15]. Due to the short half life of α-MSH, repeating the dose in the initial days following AIS has also been suggested [11]. However, its safety in AIS patients has never been tested before and thus its potential neuroprotective effects in AIS patients is currently not known.

The aim of this study was to assess the feasibility and safety of afamelanotide for the first time in AIS patients. The hypothesis was that afamelanotide would positively affect the infarcted area (core) and the ischaemic zone of tissue (penumbra) in patients with AIS, with no major adverse effects. Validating the safety of afamelanotide in AIS patients would set the course for larger trials to test its neuroprotective properties in AIS patients.

## Materials and methods

### Study design and patient population

This was a single centre, industry sponsored (CLINUVEL Pharmaceuticals), open label, non-randomised, prospective design, phase IIa trial of afamelanotide. The study was reviewed and approved by our Institutional Review Board (HREC/68,070/Alfred-2020). Eligible patients were older than 18 with limited functional disability at baseline (premorbid modified Rankin scale (mRS) < 4); had a diagnosis of first AIS; presented within 24 h of onset of symptoms; had a distal arterial vessel occlusion and relevant perfusion mismatch confirmed on imaging. Patients who underwent acute revascularisation therapies, pregnant, lactating, allergic to melanocortins, with severe hepatic or renal impairment or inability to undergo CT or MRI scans were excluded.

### Study drug

Afamelanotide 16 mg, controlled release, sterile formulation contained in a poly D,L-lactide-co-glycolide implant (SCENESSE^®^) was administered subcutaneously via an injection into the fat above the anterior portion of the iliac crest. First dose was administered on the day of recruitment (D0) and second dose was administered 24 h later (D1). The drug was administered again on D7 and D8, when the patient’s neurological deficits still persisted.

### Imaging assessments

Computed tomography (CT) of the brain followed by CT angiography from aortic arch to the vertex and CT perfusion was performed on D0. Automated perfusion maps (RAPID) were used to measure the core and penumbra (Tmax). Magnetic resonance imaging (MRI) with DWI and FLAIR sequences was performed on D3 and D9. The volume of hyperintense lesion on FLAIR sequences was measured by a prediction algorithm using an open source toolbox which has high reproducibility compared to expert manual lesion marking [20].

### Outcome measures

The primary outcome was the safety of afamelanotide in AIS patients as assessed by monitoring and recording of treatment-emergent adverse events (TEAEs), recorded regularly up to D42. The secondary outcome was the signal of efficacy as assessed clinically by using the standardised National Institutes of Health Stroke Scale (NIHSS) up to D42 and radiologically by measuring the changes in the volumes of Tmax and FLAIR lesions.

## Results

**Fig. 1:**
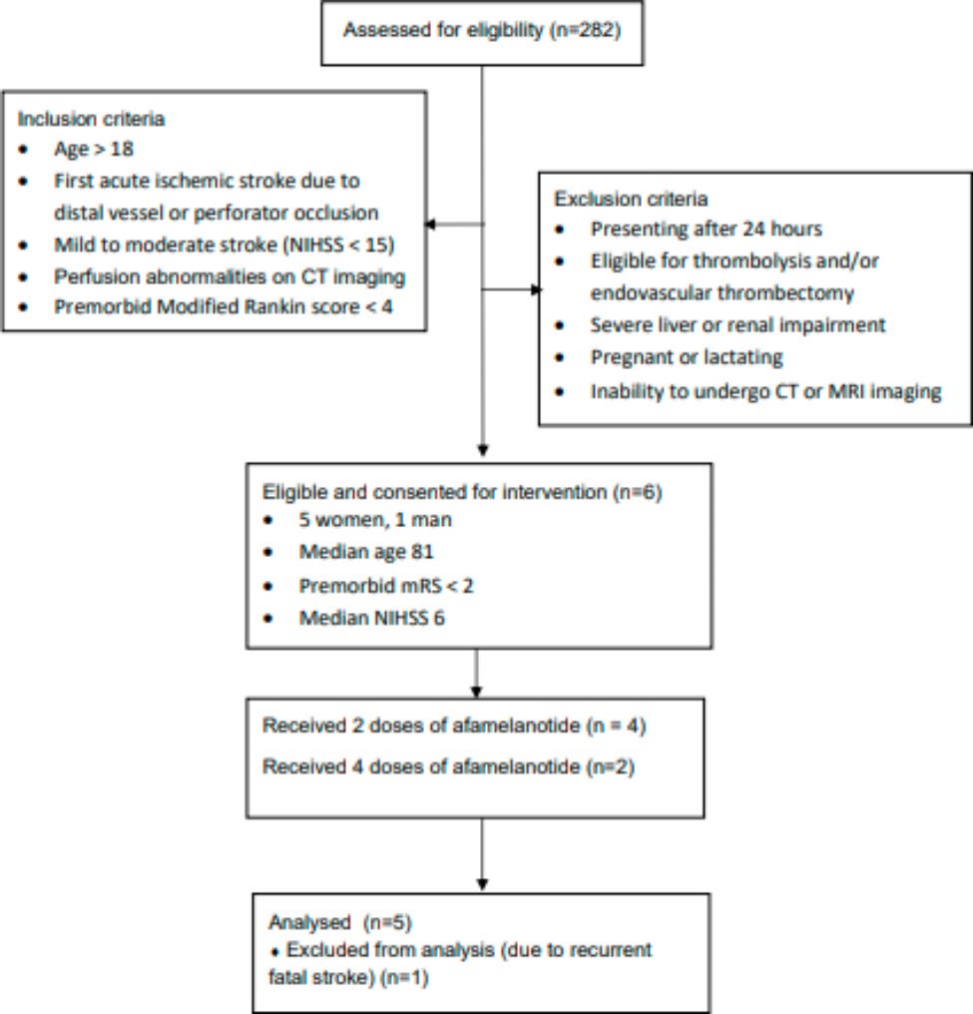
Consort diagram depicting the recruitment process

Five women and one man, with a median age of 81, all with pre-morbid mRS < 2, were recruited for the study over a period of ten months (Fig. 1). All patients had distal vessel occlusion in the middle cerebral artery and one patient also had a further occlusion in the distal posterior cerebral artery. The median NIHSS was 6 at point of study entry. The aetiology was cardioembolic in three patients, and embolic stroke of undetermined source in one patient. Two patients had internal carotid artery atherosclerosis, one with severe stenosis and one with complete occlusion. All patients received single doses of study drug on D0 and D1 and two patients had further doses on D7 and D8. The median time to treatment from stroke onset was 20 h.

### Safety

None had immediate local or major systemic TEAEs (Table 1). One patient had an asymptomatic, haemorrhagic transformation within the infarcted tissue which was assessed as Class 1c, and type PH1 (parenchymal haematoma) as per Heidelberg classification. Other TEAEs included urinary tract infection and constipation. All TEAEs were assessed as unrelated to afamelanotide and treated accordingly.

**Table 1:** Baseline demographics and adverse outcomes

Patient	Age	Sex	Stroke territory	Exclusions for acute treatment	Aetiology	Baseline NIHSS	Baseline mRS	Adverse outcomes^1^ (up to D9)
1	84	F	Left MCA	Recent hip surgery Distal clot migration	Cardioembolic	6	2	Nil
2	86	F	Left MCA	Onset > 4.5 h, on therapeutic anticoagulation Distal M2 occlusion	Cardioembolic	7	0	UTI, constipation, agitation
3	79	F	Left PCA & Right MCA	Recent hip surgery P2 occlusion	Cardioembolic	9	1	Haemorrhagic transformation (PH1), troponin rise, fluid overload
4	74	M	Left MCA	Fluctuating symptoms M2 occlusion	LAA	4	0	Nausea, loss of appetite, ankle pain, constipation
5	83	F	Left MCA	Unclear time of onset Unfavourable mismatch ratio	ESUS	5	0	Delirium
6	50	F	Left MCA	Onset > 4.5 h M2 occlusion	LAA	1	0	Recurrent stroke on D5, possible UTI, fatal haemorrhagic transformation on D9

^1^ Assessed as unrelated to afamelanotide. MCA – middle cerebral artery; PCA – posterior cerebral artery; LAA – large arterial atherosclerosis; ESUS – embolic strokes of unknown source; UTI – urinary tract infection; PH1 – Parenchymal haematoma 1

One patient who had complete carotid occlusion and NIHSS of 1 on D0 suffered a recurrent ischemic stroke on D5 with an increase in NIHSS to 13. Repeated imaging showed propagation of distal M2 thrombus proximally to M1, resulting in long segment MCA occlusion and associated new infarction, with subsequent fatal haemorrhagic transformation (Class 2, PH2) on D9. This patient received only two doses of study drug, on D0 and D1. The recurrent stroke was assessed as unrelated to the study drug.

### Efficacy Signal

The median NIHSS of all surviving patients was improved from 6 on D0 to 2 on D7 and to 1 on D42 (Table 2). On D42, all patients were living at home and functionally independent with an mRS < 3.

**Table 2:** Stroke deficits as assessed by NIHSS

Patient	Baseline/D0	D1	D2	D3	D4	D7	D8	D42
1	6	2	3	2	4	0	0	NP
2	7	6	5	6	NP	5	5	3
3	9	6	3	NP	NP	3	3	1
4	4	2	NP	NP	NP	2	NP	1
5	5	4	NP	NP	NP	1	NP	0
6*	1	2	2	2	2	13	NP	NP
Median	6	4				2		

NP – Not performed. * - not included in median NIHSS

**Fig. 2:**
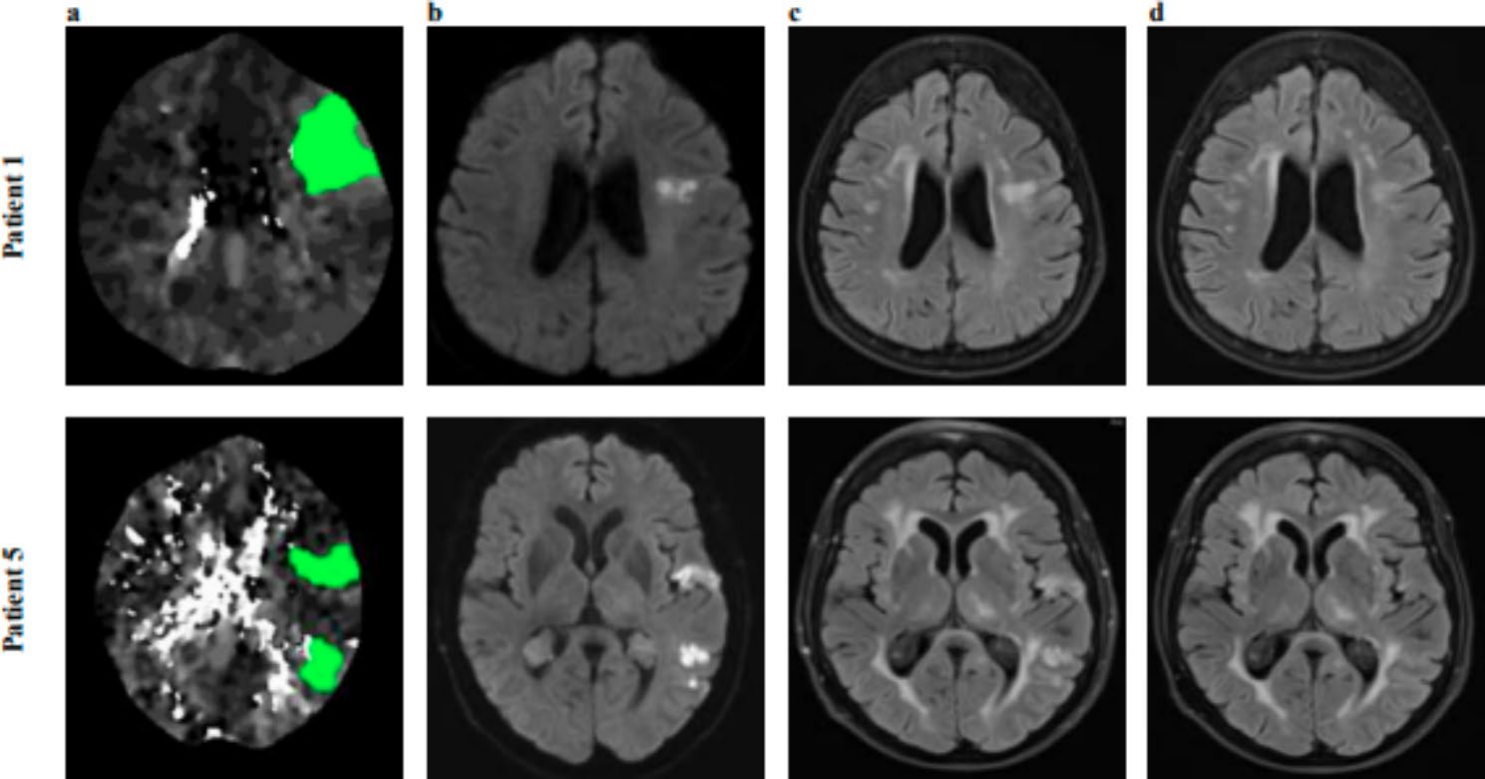
Tmax images on D0 (**a**) with relevant DWI (**b**) and FLAIR lesions on D3 (**c**) and FLAIR lesions on D9 (**d**) from patients 1 and 5

**Fig. 3:**
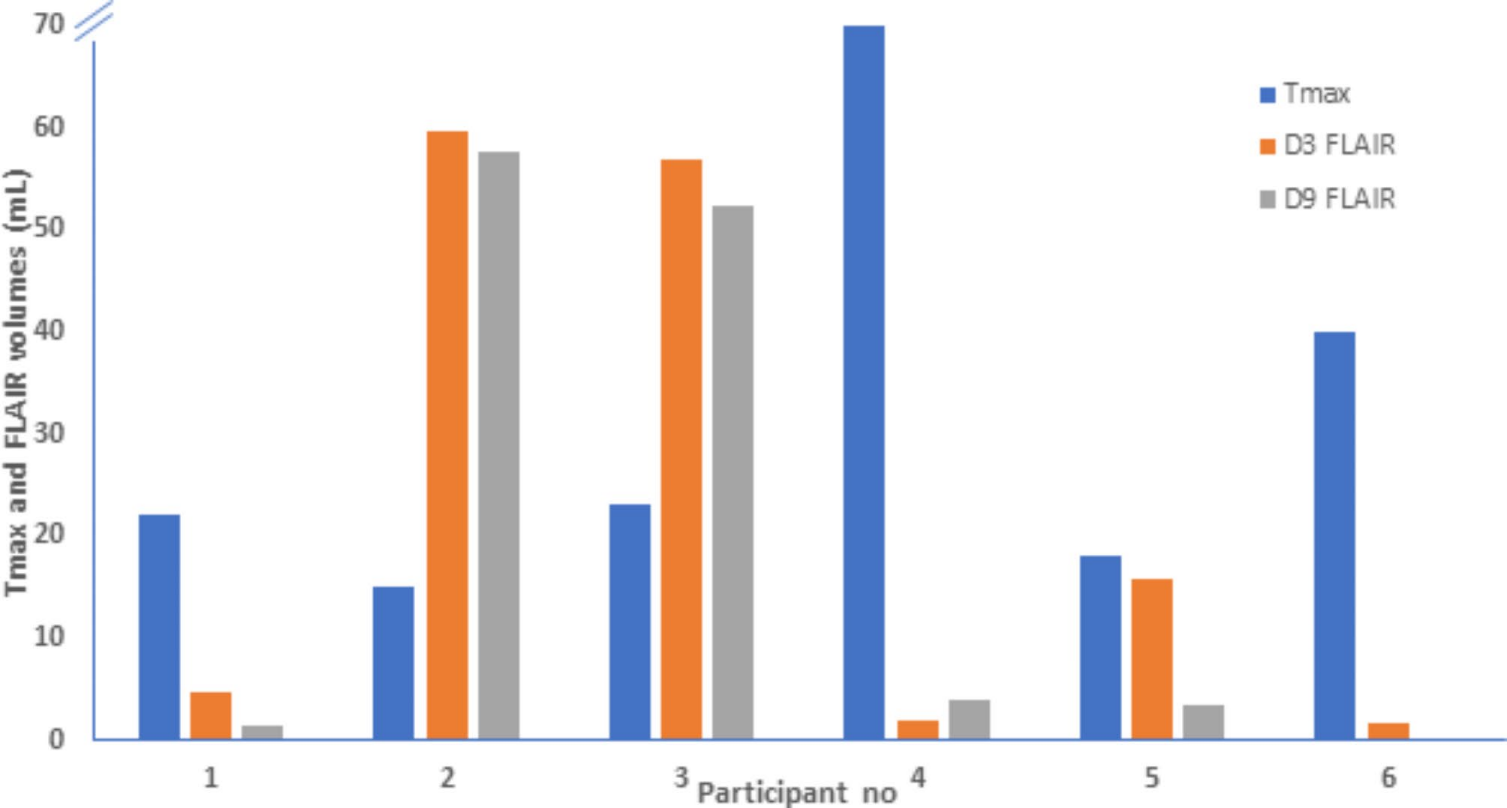
Comparison of Tmax and FLAIR volumes for all patients

Single slice samples of Tmax images on D0 and FLAIR sequences on D3 and D9 for 2 patients, showing progressive reduction in acute lesions, is shown in Fig. 2. Overall, four patients showed a reduction in FLAIR volume on D3 compared to the Tmax volume on D0 while two patients had increased FLAIR volume on D3 (Fig. 3). The median Tmax volume on D0 was 23 mL which was reduced to a FLAIR volume of 10 mL on D3 and 4 mL on D9.

## Discussion

Our phase IIa clinical trial shows that administration of afamelanotide 16 mg implant in AIS patients is safe and well tolerated. This is the first time afamelanotide has been evaluated in AIS patients. Afamelanotide also appears to be associated with meaningful neurological recovery and radiological improvement of salvageable tissue.

None of our patients had any serious adverse drug reactions. Minor, transient adverse events where present, were considered to be likely unrelated to afamelanotide. The minor adverse events were also very different between patients, suggesting afamelanotide was an unlikely cause. Afamelanotide is currently licenced in United States, Europe and Australia for treatment of EPP. Post marketing surveillance over several years has confirmed the positive safety profile of afamelanotide with no significant drug related adverse effects [18, 21, 22]. Implant site reaction and nausea were the most common side effects with incidence rate of up to 21% [23].

Four of six patients showed reduction in the radiological measurements of infarct core on D3 and all surviving patients showed improvement in NIHSS at 42 days. Although the measurements of CT perfusion on D0 and MRI-FLAIR on D3 are not directly comparable, the direction and magnitude of reduction in infarct core volume was clearly evident. A repeat CT perfusion on D3 would risk unnecessary radiation and contrast related adverse effects while an MRI on D0 is not always practical. The reduction in core volume was also despite of a median time to treatment from stroke onset of 20 h. Neuroprotective agents are likely to result in better outcomes when given early in the process of ischemic damage and inflammation. However our data suggest that even at a median of 20 h, afamelanotide may provide benefit. A broad therapeutic window and longer lasting treatment effect with α-MSH hormone on AIS had been demonstrated in animal studies [15, 24].

The benefit of afamelanotide in AIS is likely due to its neuroprotective properties on the BBB. Neuroprotective agents, targeting tight junctions of BBB may confer vascular protection during AIS and other brain injuries [16, 25, 26]. Administration of α-MSH has been shown to restore the integrity of BBB in neuroinflammatory disorders [27]. The neuroprotective benefits of afamelanotide in AIS have also been tested in animal studies previously and showed reduction in final infarct volume [13, 24].

Afamelanotide may also have positive effects on AIS and other brain injuries through a direct neuromodulatory and neurotropic effect on melanocortin receptors [11, 28] giving it an unique multi-modal action in AIS. Current evidence shows that plasma α-MSH levels decrease following severe AIS and other acute brain injuries [10–12] and that exogenous administration of α-MSH following AIS improve stroke outcomes [11]. Maintenance of plasma α-MSH after stroke may therefore not only be protective to the penumbra and BBB but can also enhance neuroplasticity mechanisms, and thus can extend the time window for treatment effect [11, 16].

### Strengths and limitations

Our study is the first ever that applied afamelanotide in AIS patients. The patient population was highly characterised based on strict inclusion criteria and multimodal CT imaging. All the patients were treated under 24 h.

Limitations in this study include a small study population with gender disproportion. Although no serious adverse reactions were encountered in this small phase IIa feasibility study, a larger sample size is required to draw reliable conclusions regarding afamelanotide’s safety and signal of efficacy. All our patients had NIHSS less than 9, suggesting mild to moderate stroke. It can be argued that the changes seen in NIHSS with time was likely due to the natural recovery from stroke, however this may not explain the radiological changes and randomized placebo controlled trials are warranted.

In summary, afamelanotide was safe, well tolerated and showed possible reduction in infarct core volume in our safety and feasibility study involving small sample of AIS patients. Potent MSH analogues such as afamelanotide have high therapeutic potential in AIS. Further large, randomized studies are required.

## Data Availability

All data generated or analysed during this study are included in this published article.

## References

[1] Feigin VL, Stark BA, Johnson CO, Roth GA, Bisignano C, Abady GG (2021). Global, regional, and national burden of stroke and its risk factors, 1990–2019: a systematic analysis for the global burden of Disease Study 2019. Lancet Neurol.

[2] Michel P, Diepers M, Mordasini P, Schubert T, Bervini D, Rouvé JD (2021). Acute revascularization in ischemic stroke: updated swiss guidelines. Clin Translational Neurosci.

[3] O’Collins VE, Macleod MR, Donnan GA, Horky LL, van der Worp BH, Howells DW (2006). 1,026 experimental treatments in acute stroke. Ann Neurol.

[4] Goenka L, Uppugunduri Satyanarayana CR, George SSK (2019). Neuroprotective agents in Acute ischemic Stroke—A reality check. Biomed Pharmacother.

[5] Green AR (2008). Pharmacological approaches to acute ischaemic stroke: reperfusion certainly, neuroprotection possibly: pharmacological approaches to acute ischaemic stroke. Br J Pharmacol.

[6] Ginsberg MD (2008). Neuroprotection for ischemic stroke: past, present and future. Neuropharmacology.

[7] Holloway PM, Durrenberger PF, Trutschl M, Cvek U, Cooper D, Orr AW (2015). Both MC _1_ and MC _3_ receptors provide Protection from Cerebral Ischemia-Reperfusion–Induced Neutrophil Recruitment. ATVB.

[8] Holloway PM, Smith HK, Renshaw D, Flower RJ, Getting SJ, Gavins FNE (2011). Targeting the melanocortin receptor system for anti-stroke therapy. Trends Pharmacol Sci.

[9] Tatro JB (2006). Melanocortins defend their Territory: multifaceted neuroprotection in cerebral ischemia. Endocrinology.

[10] Zierath D, Tanzi P, Cain K, Shibata D, Becker K (2011). Plasma α-melanocyte stimulating hormone predicts outcome in ischemic stroke. Stroke.

[11] Savos AV, Gee JM, Zierath D, Becker KJ (2011). α-MSH: a potential neuroprotective and immunomodulatory agent for the treatment of stroke. J Cereb Blood Flow Metab.

[12] Magnoni S, Stocchetti N, Colombo G, Carlin A, Colombo A, Lipton JM (2003). α-Melanocyte-stimulating hormone is decreased in plasma of patients with Acute Brain Injury. J Neurotrauma.

[13] Chen G, Frøkiær J, Pedersen M, Nielsen S, Si Z, Pang Q (2008). Reduction of ischemic stroke in rat brain by alpha melanocyte stimulating hormone. Neuropeptides.

[14] Giuliani D, Leone S, Mioni C, Bazzani C, Zaffe D, Botticelli AR (2006). Broad therapeutic treatment window of [Nle4, D-Phe7]α-melanocyte-stimulating hormone for long-lasting protection against ischemic stroke, in mongolian gerbils. Eur J Pharmacol.

[15] Giuliani D, Mioni C, Altavilla D, Leone S, Bazzani C, Minutoli L (2006). Both early and delayed treatment with melanocortin 4 receptor-stimulating Melanocortins produces neuroprotection in cerebral ischemia. Endocrinology.

[16] Bitto A, Polito F, Irrera N, Calò M, Spaccapelo L, Marini HR (2012). Protective effects of melanocortins on short-term changes in a rat model of traumatic brain injury*. Crit Care Med.

[17] Forslin Aronsson Ã, Spulber S, Popescu LM, Winblad B, Post C, Oprica M (2006). α-Melanocyte-stimulating hormone is neuroprotective in rat global cerebral ischemia. Neuropeptides.

[18] Biolcati G, Marchesini E, Sorge F, Barbieri L, Schneider-Yin X, Minder EI (2015). Long-term observational study of afamelanotide in 115 patients with erythropoietic protoporphyria. Br J Dermatol.

[19] Biolcati G, Deybach JC, Hanneken S, Wilson P, Wahlin S, Varigos G et al. A randomized phase III trial of afamelanotide (Scenesse (R)), an agonistic alpha-melanocyte stimulating hormone analogue in the treatment of protoporphyria-induced phototoxicity. In: Br J Dermatol. 2011. p. 1143–3.

[20] Egger C, Opfer R, Wang C, Kepp T, Sormani MP, Spies L (2017). MRI FLAIR lesion segmentation in multiple sclerosis: does automated segmentation hold up with manual annotation?. Neuroimage Clin.

[21] Wensink D, Wagenmakers MAEM, Barman-Aksözen J, Friesema ECH, Wilson JHP, van Rosmalen J (2020). Association of afamelanotide with improved outcomes in patients with erythropoietic protoporphyria in clinical practice. JAMA Dermatol.

[22] Minder AE, Barman-Aksoezen J, Schmid M, Minder EI, Zulewski H, Minder CE (2021). Beyond pigmentation: signs of liver protection during afamelanotide treatment in swiss patients with erythropoietic protoporphyria, an observational study. Therapeutic Adv Rare Disease.

[23] Langendonk JG, Balwani M, Anderson KE, Bonkovsky HL, Anstey AV, Bissell DM (2015). Afamelanotide for erythropoietic protoporphyria. N Engl J Med.

[24] Giuliani D, Leone S, Mioni C, Bazzani C, Zaffe D, Botticelli AR (2006). Broad therapeutic treatment window of [Nle4, D-Phe7]α-melanocyte-stimulating hormone for long-lasting protection against ischemic stroke, in mongolian gerbils. Eur J Pharmacol.

[25] Wu X, Fu S, Liu Y, Luo H, Li F, Wang Y (2019). NDP-MSH binding melanocortin-1 receptor ameliorates neuroinflammation and BBB disruption through CREB/Nr4a1/NF-κB pathway after intracerebral hemorrhage in mice. J Neuroinflammation.

[26] Rinne P, Nordlund W, Heinonen I, Penttinen AM, Saraste A, Ruohonen ST (2013). α-Melanocyte-stimulating hormone regulates vascular NO availability and protects against endothelial dysfunction. Cardiovascular Res.

[27] Mykicki N, Herrmann AM, Schwab N, Deenen R, Sparwasser T, Limmer A (2016). Melanocortin-1 receptor activation is neuroprotective in mouse models of neuroinflammatory disease. Sci Transl Med.

[28] Namba K, Kitaichi N, Nishida T, Taylor AW (2002). Induction of regulatory T cells by the immunomodulating cytokines alpha-melanocyte-stimulating hormone and transforming growth factor-beta2. J Leukoc Biol.

